# Membrane Type-1 Matrix Metalloproteinase Expression in Acute Myeloid Leukemia and Its Upregulation by Tumor Necrosis Factor-α

**DOI:** 10.3390/cancers4030743

**Published:** 2012-07-25

**Authors:** Leah A. Marquez-Curtis, Neeta Shirvaikar, A. Robert Turner, Imran Mirza, Amir Surmawala, Loree M. Larratt, Anna Janowska-Wieczorek

**Affiliations:** 1 Canadian Blood Services R&D, Edmonton, Alberta T6G 2R8, Canada; 2 Departments of Medicine and Oncology, University of Alberta, Edmonton, Alberta T6G 2G3, Canada; 3 Department of Laboratory Medicine and Pathology, University of Alberta, Edmonton, Alberta T6G 2B7, Canada

**Keywords:** acute myeloid leukemia, membrane type 1-matrix metalloproteinase, matrix metalloproteinase-2, tumor necrosis factor-α

## Abstract

Membrane type-1 matrix metalloproteinase (MT1-MMP) has been implicated in tumor invasion, as well as trafficking of normal hematopoietic cells, and acts as a physiologic activator of proMMP-2. In this study we examined MT1-MMP expression in primary acute myeloid leukemia (AML) cells. Because tumor necrosis factor (TNF)-α is known to be elevated in AML, we also investigated the effect of TNF-α on MT1-MMP expression. We found (i) MT1-MMP mRNA expression in 41 out of 43 primary AML samples tested; (ii) activation of proMMP-2 in co-cultures of AML cells with normal bone marrow stromal cells; and (iii) inhibition of proMMP-2 activation and trans-Matrigel migration of AML cells by gene silencing using MT1-MMP siRNA. Moreover, recombinant human TNF-α upregulated MT1-MMP expression in AML cells resulting in enhanced proMMP-2 activation and trans-Matrigel migration. Thus, AML cells express MT1-MMP and TNF-α enhances it leading to increased MMP-2 activation and most likely contributing to the invasive phenotype. We suggest that MT1-MMP, together with TNF-α, should be investigated as potential therapeutic targets in AML.

## 1. Introduction

Matrix metalloproteinases (MMPs) comprise a family of Zn^2+^-binding, Ca^2+^-dependent endopeptidases whose expression and activity are upregulated in most cancers [1,2,3,4,5]. Collectively, they have the capacity to degrade virtually every component of the extracellular matrix (ECM) [6,7]. Most MMPs are secreted into the extracellular milieu, except for six membrane-type (MT1–MT6)-MMPs, which are anchored to the cell surface via a type I trans-membrane segment or through glycosylphosphatidylinositol [1]. Under steady-state physiological conditions, the expression of MMPs in most tissues is relatively low, but it can increase dramatically under conditions of hypoxia and inflammation [3] and is regulated at the transcriptional level by cytokines and growth factors [8]. Previously we showed that tumor necrosis factor (TNF)-α a naturally occurring cytokine involved in normal inflammatory and immune responses, had the most pronounced stimulatory effect on the secretion of MMP-2 by normal hematopoietic stem/progenitor cells (HSPC) [9]. MMP-2 is synthesized as a zymogen (proMMP-2). Activation of proMMP-2 involves binding of MT1-MMP to tissue inhibitor of metalloproteinases (TIMP)-2, and the resulting binary complex acts as a receptor for proMMP-2, which is then cleaved by a second MT1-MMP molecule generating a fully active MMP-2 [10,11]. MT1-MMP not only activates proMMP-2, but also degrades ECM macromolecules (collagen I, II and III, gelatin, laminins 1 and 5, fibronectin, vitronectin, and aggrecan) by itself, releases cytokines and chemokines, and cleaves adhesion signalling receptors [12,13,14,15].

Previously we demonstrated that HSPC secrete soluble MMPs, such as MMP-2 and MMP-9 after stimulation with granulocyte-colony stimulating factor (G-CSF), and others demonstrated MMP-9 secretion by G-CSF-stimulated neutrophils and suggested the role of MMP-9 in HSPC mobilization [9,16,17]. MT1-MMP is also upregulated in HSPC following treatment with G-CSF, and we and others postulated that MT1-MMP facilitates the egress of HSPC from the bone marrow (BM) to peripheral blood (PB) [18,19,20].We and others also reported that leukemic blasts from BM or PB of acute myeloid leukemia (AML) patients secrete soluble MMP-2 and MMP-9 and proposed that these MMPs play a role in leukemic dissemination [21,22,23,24,25].

AML is a genetically heterogeneous malignancy characterized by an uncontrolled proliferation and accumulation of immature progenitors in the BM, their premature egress to the PB, and resultant dissemination into peripheral tissues. TNF-α is a pleiotropic cytokine involved in the pathogenesis of several immune disorders and hematological malignancies, including AML; a high serum TNF-α level is an adverse prognostic factor for survival in AML [26]. In this work we investigated the expression of MT1-MMP in AML cells and the effect of TNF-α on it. We hypothesized that TNF-α upregulates MT1-MMP in AML cells, facilitating their egress from the BM.

## 2. Experimental Section

### 2.1. Patients and Cells

PB from normal donors and patients diagnosed with AML, and leukapheresis products (from patients with lymphomas in remission without BM involvement) were collected after informed consent and in accordance with the University of Alberta Health Research Ethics Board guidelines. The clinical characteristics of 43 AML patients (26 male/17 female; mean age: 54 ± 16.8 years; mean% blasts in PB: 60.1%) are presented in Table 1. Light-density mononuclear cells (MNC) were separated using Ficoll-Paque PLUS (GE Healthcare, Baie d’Urfe, QC, Canada) and positively selected for CD34^+^ cells using immunomagnetic separation (Miltenyi Biotec, Auburn, CA, USA) as previously described [9]. The cells were washed with Iscove’s Modified Dulbecco’s Medium (IMDM, Invitrogen, Burlington, ON, Canada), resuspended in serum-free media, and incubated at a concentration of 2 × 10^6^ cells/mL at 37 °C in 5% CO_2_ for 48 h. For some experiments cells were treated with 20 ng/mL recombinant human (rh) TNF-α (Peprotech, Rocky Hill, NJ, USA). The cell-conditioned media were collected and evaluated by zymography or stored at −20 °C for ELISA. The cell pellets were used for RNA isolation or lysed and analyzed by Western blot. The human myelomonocytic leukemia cell line THP-1 was obtained from the American Type Culture Collection (Rockville, MD, USA) and grown in RPMI 1640 (Invitrogen) supplemented with 10% bovine growth serum (BGS, Hyclone, ThermoFisher Scientific, Nepean, ON, Canada). Colony forming unit-fibroblast (CFU-F) cultures were established from normal BM buffy coat cells as described [27]. Human umbilical vein endothelial cells (HUVEC) were cultured on gelatin-coated flasks in media consisting of M199, 10 mM L-glutamine, 250 IU/mL penicillin-streptomycin, 20% fetal calf serum (all from Invitrogen), and endothelial cell growth supplement (Collaborative Biomedical, Bedford, MA, USA). CFU-F and HUVEC were grown to sub-confluence, trypsinized, and plated in 24-well plates (1 × 10^5^ cells/mL). For co-culture experiments, PB MNC (2 × 10^6^ cells/mL) were seeded onto stromal monolayers, stimulated or not (control) with 20 ng/mL rh TNF-α and incubated for 48 h at 37 °C and 5% CO_2_. Cell-conditioned media were evaluated by zymography.

### 2.2. Gel-Based and Real-Time RT-PCR Analysis

Total RNA from AML PB MNC was extracted using TRIZOL (Invitrogen) according to the manufacturer’s instructions. RT-PCR reactions were carried out for MT1-MMP as described previously using GAPDH as internal control [28]. PCR products were electrophoresed through 2% agarose gels containing ethidium bromide and visualized under UV light using the FluorChem Imaging System; densitometric analysis was carried out using AlphaEase FCr image analysis software (Alpha Innotech, San Leandro, CA, USA). The relative level of target mRNA was regarded as the ratio between the intensities of the target primer and GAPDH bands. Real-time RT-PCR was performed as previously described [28]. Quantitative assessment of MT1-MMP mRNA levels was performed using an ABI PRISM^®^ 7000 Sequence Detection System (ABI, Foster City, CA, USA) and primer (Quantitect Hs_MMP14_1_SG) from Qiagen (Toronto, ON, Canada). A 25 μL reaction mixture containing 12.5 μL Quantifast SYBR Green PCR master mix (Qiagen) and 10 ng of cDNA template, forward and reverse primers, were used. The threshold cycle (Ct), *i.e*., the cycle number at which the amount of amplified gene of interest reached a fixed threshold, was subsequently determined. Relative quantitation of MT1-MMP mRNA expression was calculated using the comparative Ct method. The relative quantitation value of target, normalized to an endogenous control gene and relative to a calibrator, is expressed as 2-∆∆Ct (fold difference), where ∆Ct = (Ct of target gene [MT1-MMP]) – (Ct of endogenous control gene), and ∆∆Ct = (∆Ct of samples for target gene) – (∆Ct of calibrator for the target gene).

### 2.3. Western Blot

Cell pellets were sonicated in lysis buffer (1% Triton, 10 mM Tris-HCl, 150 mM NaCl, 1 mM EDTA, 1 mM EGTA, 0.5 mM phenylmethylsulphonyl fluoride, 1 mM Na_3_VO_4_, 5 μg/mL leupeptin, 5 μg/mL aprotinin, and 0.5 μg/mL pepstatin A, pH 7.3; all reagents from Sigma, Oakville, ON, Canada) and the lysates clarified by centrifugation at 18,000 × *g* for 10 min at 4 °C. Protein concentration was determined using the Bio-Rad protein assay according to the manufacturer’s instructions (Bio-Rad, Hercules, CA, USA), and equal amounts of protein (30 μg) were loaded in each well. Samples were resolved in a 10% polyacrylamide gel under reducing conditions and transferred to a nitrocellulose membrane. Following blockage with 5% fat-free dried milk in Tris-buffered saline and 0.05% Tween 20, the membrane was probed with rabbit anti-human MT1-MMP polyclonal antibody (Abcam, Cambridge, MA, USA) and a secondary antibody (goat anti-rabbit, HRP-conjugated IgG, Pierce Biotechnology, Rockford, IL, USA). Chemiluminescence detection was performed using the Supersignal West Pico system (Pierce). The intensity of the bands was analyzed by densitometry using the AlphaEase FCr image analysis software (Alpha Innotech).

### 2.4. Trans-Matrigel Invasion Assay

Cell invasion was determined using the trans-Matrigel assay as we described previously [9,29]. Briefly, 13 mm polycarbonate filters (Costar/Nucleopore, Toronto, ON, Canada) were coated with 25 μg of Matrigel (Collaborative Biomedical Products) and placed between the upper and lower compartments of modified Boyden chambers (Neuro Probe Inc, Gaithersburg, MD, USA). The lower chambers contained IMDM supplemented with 0.1% BSA. AML PB MNC treated or not (control) with 20 ng/mL rh TNF-α were loaded onto the upper compartments (2 × 10^5^ cells/chamber) and incubated overnight at 37 °C, 5% CO_2_. In some experiments MNC were pre-incubated (1 h) with various concentrations of (−)-epigallocatechin 3-gallate (EGCG, Sigma) or 1 μg/mL Enbrel (Amgen, Missisauga, ON, Canada) before rh TNF-α treatment (2 h). Cells that had migrated through the Matrigel-coated filters were recovered from the lower compartments and counted using a Neubauer hemocytometer. Percentage migration was calculated from the ratio of cells recovered from the bottom chambers to the total number of cells loaded. The assay was performed at least in quadruplicate for each condition and each sample tested.

**Table 1:** Clinical characteristics of AML patients.

Pt #	Age/Sex	Diagnosis (WHO Classification)	Karyotype	Hb g/L	Plt 10^9^/L	WBC 10^9^/L	% Blasts in PB	% Blasts in BM	MT1-MMP
1	23/M	AML with inv(16)	46,XY,inv(16)(p13.1q22)[16]	135	47	179	70	63	+
2	22/M	AML without maturation	51~54,XY,+Y,+4,+8,+10,+13,+20,+21,+22[CP20]	77	19	166	87	94	+
3	75/M	Acute myelomonocytic leukemia	46,XY,del(20)(q11.2)[6]/47,idem,+8[6]/~45,idem,+8,−10[cp2]	100	28	80.9	65	62	-
4	44/F	Acute myelomonocytic leukemia	46,XX[20]	78	98	152.4	67	62	+
5	58/M	AML without maturation	46,XY[20]	144	33	59.8	73	83	+
6	23/M	AML with t(15:17)	46,XY,t(15;17)(q22;q11)[6]/48,XY,der(9;15)t(9;15;17)(q22;q21),ider (17)(q10)t(15;17)(q22;q11),+ider (17)(q10)t(15;17)(q22;q21),+21[13]	96	11	5.2	38	67	+
7	66/F	Acute myelomonocytic leukemia	46,XX[19]	99	55	15.1	50	72	-
8	37/F	Acute monoblastic leukemia	48,XX,+8,+8[14]/96,idemx2[2]/46,XX[1]	121	190	91.8	84	81	+
9	17/M	Acute myelomonocytic leukemia	46,XY[20]	52	27	101.2	44	51	+
10	78/M	AML without maturation	46,XY[20]	104	58	101.4	81	88	+
11	36/M	AML with t(15:17)	46,XY,t(17;19;22)(q21;p13;q13).nuc ish 15q22(PMLx2),17q21(RARAx2)(PML con RARAx1)[225]	143	18	94	89	92	+
12	52/M	Acute myelomonocytic leukemia	46,XY[20]	89	121	22.7	18	23	+
13	61/F	AML with t(15:17)	15q22(PMLx2),17q21(RARAx2)(PML con RARAx1)[231]	83	49	33.6	27.9	83	+
14	28/M	AML with t(15:17)	46,XY,t(15;17)(q22;q21.1)[2]/46,XY,t(15;17)(q22;q21.1),add(20)(p?11.2)[18]	91	21	21.6	84	77	+
15	49/F	AML without maturation	46,XX[20]	126	17	145.8	94	90	+
16	38/M	Acute myelomonocytic leukemia	46,XY[20]	64	87	44.1	49	59	+
17	46/M	Acute monocytic leukemia	46,XY[20]	92	16	66.7	58	89	+
18	77/M	AML with t(8;21)	45,XY,−Y,t(8;21)(q22;q22)[10]	67	69	6.3	62	51	+
19	62/M	AML with 11q23 abnormality	46,XY,del(11)(q23)	123	60	71.2	80	82	+
20	57/M	AML with multilineage dysplasia	43,X,−Y[20],−3[20],−5[20],−[20]+8[3],der(3)t(3;13)(q21;q34)[20]add(17)(p11.2)[20],+mar[4][cp20]	104	31	15.2	52	60	+
21	48/F	Acute monocytic leukemia	46,XX[20]	105	120	7.1	54	79	+
22	50/M	Acute monocytic leukemia	46,XY[25]	140	78	109.9	67	91	+
23	67/M	Biphenotypic acute leukemia	45~47,XY,−2[7],−7[6], add(11)(q23)[5],−12 [4], +1~7mar[6] [cp6]/46, XY[1] nuc ish 11q23 (MLLstx2) (91.0%)	98	72	2.5	13	49	+
24	72/M	AML NOC	ND	84	39	207	87	64	+
25	72/M	AML NOC	43,XY,−3,−7,der(8)t(8;15)(p23;q15), −9,−9,−13,−13,−15,−16, add(17)(p11.2),der(20)t(3;20) (q13.2;q13.1),dic(21,?)(q21;?),der (22)t(3;22)(p23;q13),−16,add(17) (p11.2),der(20)t(3;20) (q13.2;q13.1),dic(21;?)(q21;?), der(22)t(3;2)(p23;q13),+mar1, +mar2,+mar3,+mar4,+mar5, +mar6[3]	87	8	10	25	22	+
26	75/F	AML with maturation	44,XX,del(5)(q15q31),−7,−18[18]/45,sl,+9[3]	119	46	5.8	36	33	+
27	47/M	AML without maturation	45,XY,add(2)(p11.2),del(5)(q13q15),del(9)(q22),add(11)(p15),−17[19]	92	21	19.3	83	62	+
28	61/F	Relapsed Acute myelomonocytic leukemia	46,XX[20]	85	37	112.6	87	73	+
29	54/F	AML with inv(16)	46,XX,inv(16)(p13q22)[18]	119	46	21	36	40	+
30	37/F	AML with maturation	46,XX[20]	83	42	188.5	91	77	+
31	60/F	AML with t(8;21)	45,X,−X,t(8;21)(q22;q22)[18]/46, XX[2]	88	44	8.8	65	66	+
32	74/F	AML without maturation	47,XX,+13[11]/46,sl,−X[4]	114	28	29.1	95	93	+
33	45/M	Relapsed AML with t(15;17)	46,XY,t(15;17)(q22;q21)[10]/46,XY,idem,del(9)(q13q22)[6]/46,XY[2]	116	18	10.0	60	79	+
34	69/M	AML with multilineage dysplasia	45,XY,−7[20]	123	14	93.9	36	35	+
35	66/F	Relapsed AML	46,X,−X,+mar[15]/46,XX[4]	106	46	3.3	21	87	+
36	66/M	AML with multilineage dysplasia	43~44,XY,−5[20],del(7)(q31)[20],+8[20],−12[20],−13[20],+add(14) (q32)[20],−17[20],18[5],der(8) t(12;18) (q13;p11.3)[20],−20[20], +mar[13],+mar1x2[5],1dmin[15][cp20]	99	19	18	30	46	+
37	68/M	AML with inv(16)	46,XY,inv(16)(p13.1q22)[19]	94	20	121.2	70	66	+
38	73/M	Relapsed AML	46,XY[19]	108	56	14.8	86	88	+
39	64/F	AML with maturation	47,XX,+4[5]/46,XX[9]	87	214	46.8	72	84	+
40	34/F	AML with maturation	46,XX,del(9)(q13q22)[20]	79	29	10.2	69	76	+
41	55/F	AML with multilineage dysplasia	46,XX[18]	105	68	39.6	23	60	+
42	66/M	AML with multilineage dysplasia	47,XY,+8[18]	99	11	79.6	88	89	+
43	50/F	Acute myelomonocytic leukemia	45,XX,inv(3)(q21q26),−7[18]	94	301	18.2	18	26	+
****	****	****	**Mean ± SD (n = 43)**	**100.3 ± 20.8**	**56.6 ± 58.0**	**61.7 ± 58.2**	**60.1 ± 24.7**	**67.8 ± 20.3**	****

### 2.5. SiRNA Electroporation

Small interfering (si)RNA was targeted against 21-nucleotide sequences of MT1-MMP (Dharmacon, Lafayette, CO, USA) as described [18,30]. A control siRNA sequence was generated from a scrambled MT1-MMP sequence. THP-1 cells (5 × 10^6^) were electroporated with siRNA oligonucleotides (5 μg) using a nucleofector kit (Amaxa, Gaithersburg, MD, USA) according to the manufacturer’s instructions and incubated for 24 h in RPMI + 10% BGS at 37 °C and 5% CO_2_. Viability of the transfected cells as determined by the trypan blue exclusion test was >80%. The efficiency of MT1-MMP knockdown was determined by flow cytometry.

### 2.6. Flow Cytometric Analysis

Cells were labelled with rabbit anti-human MT1-MMP antibody (Millipore) for 45 min at 4 °C, and then stained with goat anti-rabbit IgG-555 (Invitrogen). The cells were then fixed in 1% paraformaldehyde and analyzed by flow cytometry (FACS Calibur, Becton Dickinson, San José, CA, USA) and FCS Express V3 (De Novo Software, Los Angeles, CA, USA).

### 2.7. Zymography

The gelatinolytic activities in cell-conditioned media were assessed using sodium dodecyl sulfate-polyacrylamide gel electrophoresis as we described [9]. Briefly, conditioned media were applied onto a 12% SDS-PAGE gel containing 1.5 mg/mL gelatin (Sigma) alongside media conditioned by fibrosarcoma HT-1080 cells, which exhibit both the active and pro- forms of MMP-2 and MMP-9. Clear bands at 92 kDa, 72 kDa and 62 kDa against a Coomassie blue background indicated the presence of proMMP-9, proMMP-2 and active MMP-2, respectively.

### 2.8. Enzyme-Linked Immunosorbent Assay (ELISA)

TNF-α levels were measured by ELISA (Peprotech) in media conditioned by PB MNC from normal donors and AML patients as described above. Briefly, a 96-well plate was coated with 1 μg/mL anti-human TNF-α antibody and incubated overnight at room temperature. The next day, the wells were washed (0.05% Tween-20 in PBS) and block buffer (1% BSA in PBS) added. Standards and samples (100 μL) were loaded onto wells in triplicate, the plate was incubated overnight at room temperature, and after washing, detection antibody (0.5 μg/mL) was added. For color development, avidin peroxidase conjugate and 2,2'-azino-bis(3-ethylbenzthiazoline-6-sulphonic acid (ABTS, both from Sigma) were used, and optical was density measured at 405 nm with background correction at 650 nm.

### 2.9. Statistical Analysis

Arithmetic means and standard deviations were calculated and statistical analysis of significance was carried out using a paired, 2-tailed Student’s t-test with 95% confidence interval. Calculated *p*-values between data sets were considered significantly different when *p *≤ 0.05.

## 3. Results

### 3.1. AML Cells Highly Express MT1-MMP in Contrast to Normal Cells

**Figure 1:**
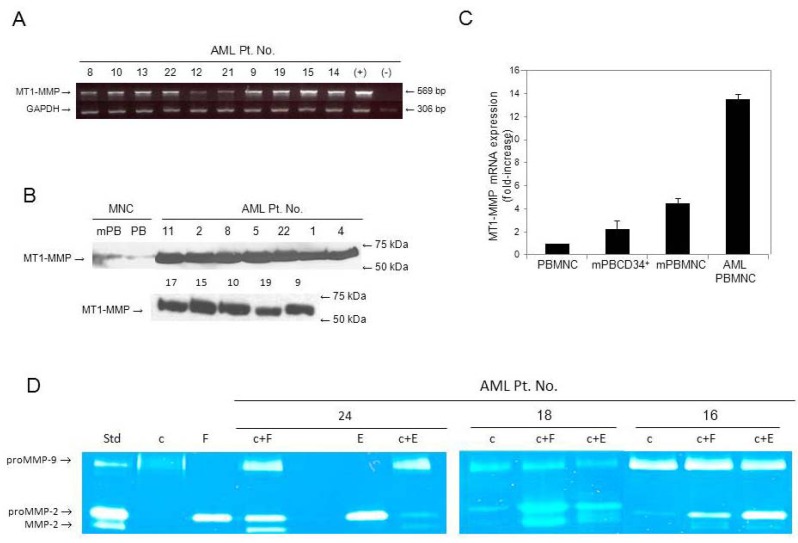
(**A**) Representative gel-based RT-PCR analysis of mRNA transcripts for MT1-MMP in MNC from patients diagnosed with AML. Patient numbers (Table 1) are shown on top of the gel along with positive (+) and negative (−) controls. GAPDH was used as the mRNA internal control to ensure equivalence of loading; (**B**) Western blot of MT1-MMP in lysates of MNC from representative AML patients; (**C**) MT1-MMP mRNA expression by real-time RT-PCR analysis of normal hematopoietic cells (MNC from normal (n = 4) and mobilized (m)PB (n = 4), and CD34+ cells (n = 2)) and MNC from AML (n = 7); (**D**) Zymogram of media conditioned by BM CFU-fibroblasts (**F**) or human umbilical vein endothelial cells (**E**), monocultures of MNC (cells only, c) from three representative AML patients, and their co-cultures with fibroblasts (c+F) or with endothelial cells (c+E). Media conditioned by fibrosarcoma HT-1080 cells were used as standard (Std) to indicate the positions of proMMP-9, proMMP-2, and active MMP-2.

Transcripts for MT1-MMP were found in 95% (41 out of 43) of AML samples tested (Table 1 and representative data in Figure 1A). MT1-MMP protein, as evaluated by Western blotting, was strongly expressed in AML cells (Figure 1B) in contrast to the weaker expression shown previously by us in normal BM MNC and CD34^+^ HSPC [18]. Here we also found, using quantitative RT-PCR analysis, that both steady-state and mobilized MNC and mobilized CD34^+^ cells from normal PB have significantly lower MT1-MMP mRNA expression compared to MNC from AML PB (Figure 1C).

### 3.2. Secretion of proMMPs by AML Cells and Active MMP-2 in Co-Cultures with Stromal Cells

Previously, we reported that AML blasts (from 15 patients diagnosed with AML) express MMP-9 and MMP-2 at the gene and protein levels [22]. Here, we extended our study to 43 additional patients diagnosed with AML (Table 1). We confirmed that AML cells strongly secrete proMMP-9 and various levels of proMMP-2, while monocultures of stromal cells (BM fibroblasts or endothelial cells) secrete only proMMP-2 [22]. Furthermore, we show here that when AML MNC were co-cultured with stromal cells, not only are the levels of proMMP-2 increased, but the active form (MMP-2) appeared (Figure 1D). Whereas active MMP-2 was undetectable in serum-free media conditioned by AML MNC alone, it was observed in co-cultures of stromal cells with MNC from two patients (#16 and #18) that weakly secrete proMMP-2, and from one (#24) that did not. Moreover, we recently reported that co-cultures of fibroblasts with normal BM CD34^+^ cells, which do not secrete proMMP-2, show active MMP-2 [18]. This suggests that proMMP-2 secreted by stromal cells can be activated by MT1-MMP secreted by hematopoietic cells and both are necessary for the proteolytic cascade leading to proMMP-2 activation [10,22].

### 3.3. MT1-MMP Inhibition Reduces Cell Migration Across Reconstituted Basement Membrane

**Figure 2:**
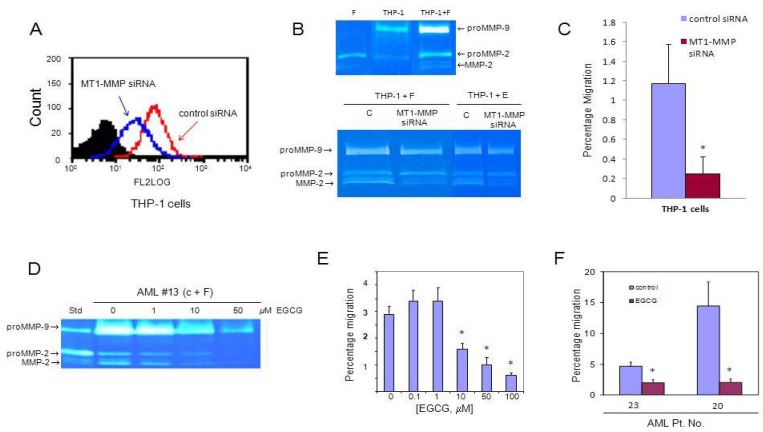
Inhibition of MT1-MMP expression and its effect on trans-Matrigel migration. (**A**) Flow cytometric analysis of MT1-MMP expression in THP-1 cells transfected with either scrambled siRNA (control) or MT1-MMP siRNA; (**B**) Zymogram of media from monocultures and co-cultures of fibroblasts and THP-1 cells pre-treated with scrambled siRNA (control, c) or MT1-MMP siRNA; (**C**) Trans-Matrigel migration of THP-1 cells transfected with scrambled siRNA (control) and MT1-MMP siRNA. The assay was performed at least in quadruplicate for each condition; (**D**) MMP activities in media conditioned by co-cultures of AML MNC from a representative patient (Pt. No. 13) and CFU-fibroblasts (**F**) in the presence of increasing doses of EGCG; (**E**) Dose-dependence of inhibition by EGCG of trans-Matrigel migration of MNC; (**F**) Percentage migration of MNC from two representative AML patients in the presence or absence (control) of 50 μM EGCG. The assay was performed at least in quadruplicate for each condition and each sample. * *p*≤ 0.05 relative to control.

We silenced the gene expression of *MT1-MMP* in leukemic THP-1 cells with siRNA. The expression of *MT1-MMP* in transfected cells was 50% of that in cells transfected with scrambled (control) siRNA (Figure 2A). We performed co-culture experiments using THP-1 cells that were pre-treated with *MT1-MMP* siRNA and found (using zymography) significantly reduced activation of proMMP-2 in conditioned media from co-cultures of these cells with fibroblasts in comparison to co-cultures with control scrambled siRNA THP-1 cells (Figure 2B). When THP-1 cells were evaluated in the trans-Matrigel migration assay, a significant reduction in migration of the *MT1-MMP* siRNA-transfected THP-1 cells was observed compared to control (scrambled siRNA) cells (Figure 2C).

Moreover, when we exposed conditioned media from co-cultures of AML cells and fibroblasts to EGCG, an inhibitor of MT1-MMP and other MMPs [31], we observed a decrease in the level of proMMP-9 and proMMP-2 as well as a dose-dependent (from 1 to 50 μM of EGCG) reduction of proMMP-2 activation (Figure 2D). EGCG had no significant effect on cell viability (data not shown). We also found that EGCG reduces the migration of AML MNC across the Matrigel in a dose-dependent manner (Figure 2E), and percentage migration was decreased by 50% to 90% using 50 μM EGCG (Figure 2F).

### 3.4. AML Cells Secrete TNF-α Which Upregulates MT1-MMP Expression and trans-Matrigel Migration

**Figure 3:**
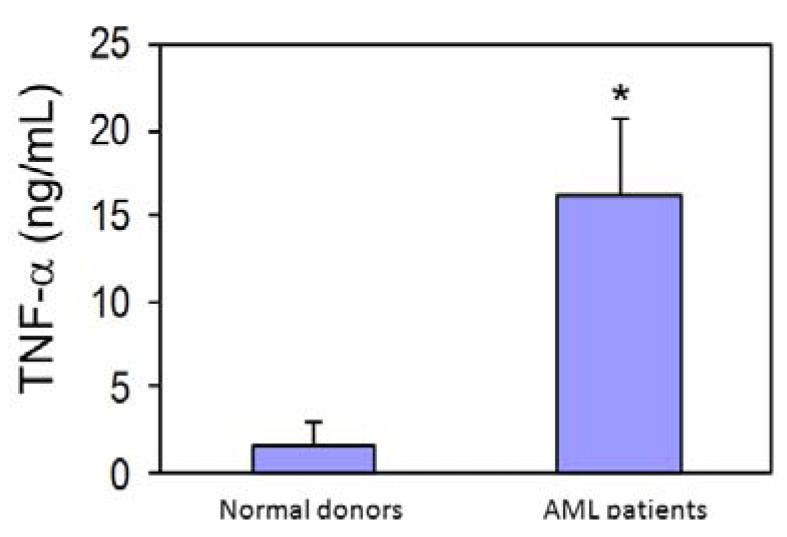
TNF-α levels in media conditioned by MNC from normal donors and AML patients as evaluated by ELISA. * *p*≤ 0.05.

Previously, we reported that rh TNF-α strongly upregulates the secretion of proMMP-2 and proMMP-9 in normal human HSPC [9]. Here using ELISA, we evaluated the level of TNF-α in media conditioned by AML MNC. We found significantly higher mean levels of TNF-α in media conditioned by AML (n = 12) compared to normal MNC (n = 3) (Figure 3).

**Figure 4:**
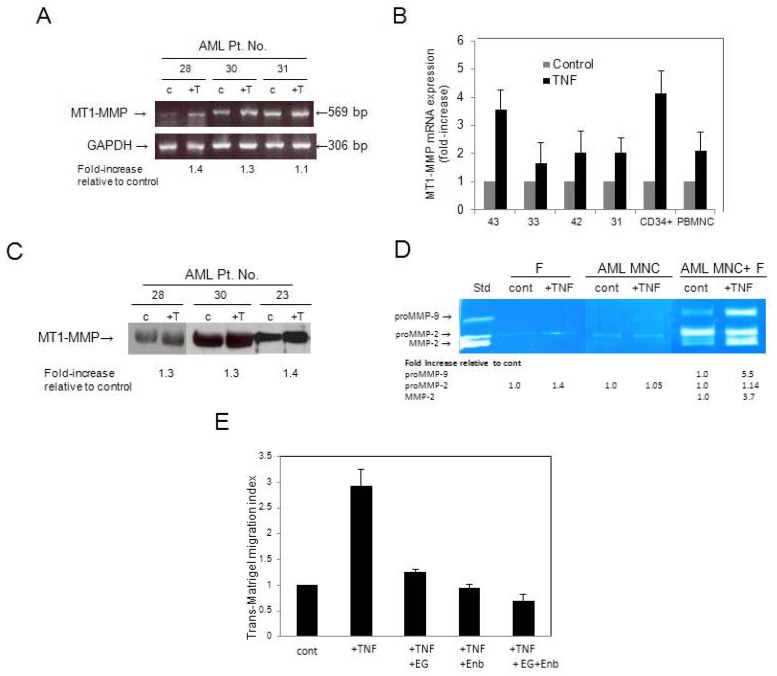
(**A**) Gel-based RT-PCR analysis of *MT1-MMP* transcript obtained from representative AML patient samples stimulated or not (control, c) with 20 ng/mL rh TNF-α (+T). *GAPDH* was used as mRNA control to ensure equivalence of loading. The numbers at the bottom of the gel represent fold-increase in expression of *MT1-MMP* relative to control as determined by densitometric analysis; (**B**) Quantitative RT-PCR analysis of *MT1-MMP* mRNA expression in MNC from representative AML patients as well as normal hematopoietic cells (CD34^+^ cells and PB MNC) stimulated or not (control) with rh TNF-α; (**C**) Western blot analysis of MT1-MMP expression in MNC from representative AML patients stimulated or not with rh TNF-α. The numbers at the bottom of the gel represent fold-increase in expression of MT1-MMP relative to control; (**D**) Zymogram of media from fibroblasts (**F**) or AML MNC with or without (control) rh TNF-α and from a co-culture of fibroblasts and MNC from a representative AML patient (Pt. No. 34), stimulated or not with rh TNF-α. Media conditioned by fibrosarcoma HT-1080 cells were used as standard (Std) to indicate the positions of proMMP-9, proMMP-2 and active MMP-2; (**E**) The TNF-α-stimulated trans-Matrigel migration of MNC from a representative AML patient was inhibited by the TNF-α-receptor fusion protein, Enbrel (Enb), and by the MT1-MMP inhibitor EGCG (EG) alone and in combination.

Having confirmed an earlier report that TNF-α is elevated in AML [26], we next examined whether TNF-α has any effect on *MT1-MMP* mRNA expression. We found that rh TNF-α increased (up to 1.4-fold by gel-based RT-PCR and up to more than 3-fold by quantitative RT-PCR) the expression of *MT1-MMP* transcripts in MNC from AML patients (Figure 4A,B, respectively). The expression of total MT1-MMP protein, as evaluated by Western blotting, was increased up to 1.4-fold (Figure 4C). The moderate increases in MT1-MMP gene and protein expression upon TNF-α stimulation could be explained by the fact that these cells had been pre-exposed to endogenously produced TNF-α. When we stimulated AML cells with rh TNF-α under co-culture conditions with fibroblasts, we observed a very strong activation of proMMP-2, although TNF-α barely affected the secretion of proMMP-2 by BM stromal fibroblasts or AML blasts in monocultures (Figure 4D). In addition, we observed that TNF-α also increased proMMP-9 secretion (5.5-fold) when AML MNC were co-cultured with fibroblasts.

Moreover, rh TNF-α stimulated the trans-Matrigel migration of AML MNC. This trans-Matrigel migration was inhibited by EGCG, as well as by Enbrel, a soluble TNF-receptor fusion protein that blocks TNF-α. Together EGCG and Enbrel had additive inhibitory effects on the trans-Matrigel migration of AML MNC stimulated with TNF-α (Figure 4E).

## 4. Discussion

Early attempts to identify MMPs responsible for mediating tumor invasion focused on soluble MMPs (particularly MMP-2 and MMP-9), and expression of these MMPs correlated with the neoplastic phenotype [32]. Previously, we and others evaluated the expression of MMP-2 and -9 and TIMP-2 in patients with AML and proposed their role in the dissemination of leukemic blasts [21,22,23,24,25,33]. Because MMP-2 and MMP-9 are synthesized as latent enzymes, here we focused on MT1-MMP, the most common physiological activator of proMMP-2. However, we found that despite the expression of both MT1-MMP and TIMP-2, which are the putative components of proMMP-2 activation, active MMP-2 was undetectable in serum-free media conditioned by AML MNC alone. Also, BM stromal cells (fibroblasts), although expressing MT1-MMP as well as TIMP-2, secrete only proMMP-2 and no active MMP-2, as reported by us previously [22]. We evaluated 43 patients diagnosed with AML and report here the expression of MT1-MMP in 41 out of 43 (95%) patients studied (Table 1). Using gene silencing with *MT1-MMP* siRNA, we demonstrated a functional link between MT1-MMP activity and *in vitro* migration of leukemic THP-1 cells across reconstituted basement membrane. Consistent with our data, it was reported that inhibition of MMP-2 and MT1-MMP by siRNA substantially impairs the migration of a highly invasive human acute monocytic leukemia cell line SHI-1 [34]. Thus, our data present further evidence for a role of MT1-MMP in the migration and dissemination of leukemic cells.

It has been shown that MT1-MMP siRNA-treated tumor cells are unable to penetrate three-dimensional collagen barriers [35]. Furthermore, using a highly selective humanized anti-catalytic MT1-MMP monoclonal antibody (DX-2400) in a MDA-MB231 xenograft model, it was demonstrated that MT1-MMP is the dominant proteolytic enzyme responsible for tumor invasion and metastasis [36]. High concentrations of MT1-MMP on the cell membranes of metastatic cells were shown by high-resolution multimodal microscopy to drive the dissemination of cancer cells into adjacent normal tissue [37]. Focal basement membrane remodelling is required to generate a conduit permissive for cell passage across ECM barriers, and the membrane-anchored MT-MMPs are suitably localized at the leading edge of migrating cells [38]. MT1-MMP is internalized in caveolae, cholesterol-rich membrane microdomains [13,39], and is readily available for rapid surface localization in invasive membrane protrusions called invadopodia [15]. Recruitment of MT1-MMP to invadopodia of tumor cells occurs during the metastatic process, where it enhances local ECM degradation and facilitates tumor cell migration and invasion. Our findings here suggest that the mechanisms by which leukemic cells disseminate may be similar to those utilized by tumor cells.

We also showed that TNF-α-stimulated AML cells co-cultured with fibroblasts are more potent activators of proMMP-2 than unstimulated cells and further support the role of the microenvironment for providing cues permissive for disease progression [40]. TNF-α is expressed on a variety of cells as a membrane-bound precursor (pro-TNF-α), which can be converted to its active form through proteolytic cleavage by several MMPs including MMP-2, MMP-9 and MT1-MMP [41,42,43]. We confirmed that TNF-α is constitutively secreted by AML cells and its levels are significantly higher in media conditioned by AML cells compared to normal controls. Consistent with these findings, TNF-α mRNA expression was reported to be higher in BM cells from *de novo* AML patients compared to normal controls [44].

The soluble TNF-α receptor fusion protein etanercept (Enbrel) acts as a competitive inhibitor that blocks the interaction of TNF-α with its cell surface receptors and consequently, activation of target cells, but had limited activity as a therapeutic agent in AML patients treated with chemotherapy [45]. As MMPs mediate TNF-α processing and secretion [41], it is possible that inhibition of these processes by MMP inhibitors could attenuate TNF-α activation and secretion. Moreover, given that TNF-α contributes to progression of several cancers [46], reducing TNF-α levels in AML using MMP inhibitors may dampen the inflammatory milieu in the BM microenvironment; however further studies are required to confirm it. The green tea catechins, such as EGCG, have been extensively investigated for their antioxidant and anti-inflammatory capabilities, particularly as chemopreventive agents by suppressing MMP expression [43]. Here we showed that a combination of the TNF-α antagonist Enbrel with EGCG diminishes the in vitro invasion of AML cells across reconstituted basement membrane by almost 80%. The widespread involvement of MMPs in physiological processes renders broad spectrum inhibitors of MMPs clinically undesirable due to inefficacy and adverse side effects [47]; however, targeting MT1-MMP, which we show here to be upregulated under inflammatory conditions (e.g., high TNF-α levels), should be further investigated.

## 5. Conclusions

In conclusion, our findings that high TNF-α levels secreted by AML cells contribute to an upregulation in MT1-MMP expression, and enhanced proMMP-2 activation leading to increased trans-Matrigel invasion by these cells indicate a potential synergistic relationship between MT1-MMP and TNF-α. As MMPs are recognized as a therapeutic target for many cancers [2,48], our work indicates that despite the karyotypic heterogeneity among AML patients, MT1-MMP expression appears to be almost ubiquitous and suggests that targeting of MT1-MMP as part of a combinatorial approach should be investigated in designing future treatments for AML.
